# An Internet-Based Radiology Course in Medical School: Comparison of Academic Performance of Students on Campus Versus Those With Absenteeism Due to Residency Interviews

**DOI:** 10.2196/mededu.8747

**Published:** 2018-05-18

**Authors:** Andrew George Alexander, Deborah Deas, Paul Eric Lyons

**Affiliations:** ^1^ Department of Medical Education School of Medicine University of California, Riverside Riverside, CA United States

**Keywords:** radiology clerkship, online education, imaging, radiology rotation, Web-based education

## Abstract

**Background:**

Imaging and its optimal use are imperative to the practice of medicine, yet many students don’t receive a formal education in radiology. Concurrently, students look for ways to take time away from medical school for residency interviewing. Web-based instruction provides an opportunity to combine these imperatives using online modalities.

**Objective:**

A largely Web-based course in radiology during the 4th year of medical school was evaluated both for its acceptance to students who needed to be away from campus for interviews, and its effectiveness on a nationally administered standardized test.

**Methods:**

All students were placed into a structured program utilizing online videos, online modules, online textbook assignments, and live interactive online lectures. Over half of the course could be completed away from campus. The Alliance of Medical Student Educators in Radiology test exam bank was used as a final exam to evaluate medical knowledge.

**Results:**

Positive student feedback included the freedom to travel for interviews, hands-on ultrasound training, interactive teaching sessions, and quality Web-based learning modules. Negative feedback included taking quizzes in-person, a perceived outdated online textbook, and physically shadowing hospital technicians. Most students elected to take the course during the interview months of October through January. The Alliance of Medical Student Educators in Radiology final exam results (70.5%) were not significantly different than the national cohort (70%) who took the course in-person. Test scores from students taking the course during interview travel months were not significantly different from students who took the course before (*P*=.30) or after (*P*=.34) the interview season.

**Conclusions:**

Students desire to learn radiology and often choose to do so when they need to be away from campus during the fall of their 4th year of study to accomplish their residency interviews. Web-based education in radiology allows students’ interview traveling and radiology course objectives to be successfully met without adversely affecting the outcomes on a nationally normed examination in radiology. A curriculum that includes online content and live Web-based teleconference access to faculty can accomplish both imperatives.

## Introduction

Imaging is an essential part of modern medicine and its proper instruction is integral to desired patient care outcomes. As important as imaging is to diagnosis, undergraduate medical education in radiology has traditionally been an elective that often occurs in a small, closed, dark room. While the images have become digital and formal interpretation of these images often occurs hundreds or thousands of miles away through the use of the internet, medical school education in radiology is generally taught in a fixed site using a single student sitting beside a single radiologist who is talking into a dictation device. These “reading sessions” slow the learning process due to the general inability to select progressive teaching cases at the level of the learner. A realistic experience in an active radiologic reading room is unfortunately limited by the random manner in which disease occurs.

Despite the necessity for radiology in most medical specialties, only 25% of medical schools require a formal education in imaging. Of the remaining schools (where radiology is not required), 63% of students express their intentions to take it as an elective [1]. Intersecting with the imperative to become fluent in modern imaging technologies, medical students frequently choose to take radiology as an elective during the months when they are away from campus interviewing for residencies [2]. Internet blogs reference “radiation vacations” as an acknowledgment that this course may offer relaxation and a chance to be away [3]. This attitude is contrary to the needs of most future physicians and further diminishes the importance of learning the use of important tools for making diagnoses.

The science of radiology is at the forefront of a digital world, but the teaching of radiology often occurs in a very “analog” manner. Since imaging technologies are now digital, they can be presented digitally to the learner. The 96.5% of graduating medical students who will not be radiologists deserve an education that emphasizes intelligent utilization of digital imaging and imaging technology [4]. These future physicians will routinely order imaging studies without consultation from radiologists. They will perform bedside ultrasound in the emergency departments and when placing central venous access catheters. Students (and their future patients) will benefit from structured medical school lessons that include appropriate didactics and clinical scenarios. Clinical judgment is enhanced by the structured review of appropriately selected experiences and supervised critical thinking. These conditions are replicated better with a structured program than with a 4-week rotation that relies heavily upon the empiric pathology of random imaging cases. The University of California, Riverside School of Medicine mandated a 4-week radiology clerkship in the 4th year of the medical school curriculum that adheres to the principles of educating new physicians in technologies of the future and that advantages the internet learning modalities of the 21st century.

## Methods

Fourth-year medical students at the University of California, Riverside enrolled in the mandatory course in radiology consisting of 20 days of instruction over 4 weeks, and optional activities on weekends. An online standard textbook [5] was used with hyperlinks given almost daily for reading assignments. Substantially more chapters were assigned in the initial weeks of the course so that students could quickly establish a foundation of knowledge upon which they may learn more efficiently. Videos from multiple online sources and a series of online modules from the website, Aquifer [6], were assigned throughout the course, with the preponderance assigned in the first 2 weeks. A copy of the curriculum is provided in Multimedia Appendix 1. Graded quizzes were given on days 1, 2, 3, 5, 8, 12, and 16 to add motivation and to assure that the Web-based didactic lessons were taken seriously. Students were introduced to bedside ultrasounds in week 2. Ultrasound teaching sessions were taught onsite at the university by non-radiology personnel who had previously passed advanced courses in Point of Care ultrasound. No online sessions with board-certified radiologists occurred until day 6, by which time the students had a basic understanding of the risks and side-effects of imaging, its indications, and the anatomy of the underlying structures. The American College of Radiology (ACR) Appropriateness Criteria for ordering imaging studies were referenced early on, and an app from the ACR was placed onto the students’ mobile (cellular) phones. Online sessions with the radiology faculty specialist were held for 2 hours on 6 different evenings. These sessions occurred over the internet using BlueJeans or Google Hangouts technology. The radiology faculty remained at a remote university and communicated with the students, both singularly, and in small groups, at sites of the students’ choice.

During the final week, each student was responsible to return to campus and present an interesting radiologic case, with digital films, to the group. On the final day, The Alliance of Medical Student Educators in Radiology (AMSER) test bank was used to administer an 80-question online final exam.

Final exam scores from the 10 different 4-week block rotations were grouped into school “trimesters.” The first 3 months (July to September), the second 4 months (October to January), and the final months (February to May) were compared. These groupings represented rational segmentation from a student’s standpoint: prior to interview season (July to September), during interview or travel season (October to January), and after interview season (February to May). A 2-tailed *t* test was used to compare the average performances of the 3 cohorts of students.

Feedback evaluation was obtained from the students at the end of each monthly rotation in radiology. Student feedback was consistently documented and tabulated from multiple students over the course of multiple months.

## Results

Students rated highly the hands-on ultrasound training and enjoyed the flexibility of being able to do their work online while away interviewing for residency positions. There was consistent praise for the quality of the Aquifer CORE series of online modules as a teaching tool. Online teaching sessions using either Google Hangouts or BlueJeans were highly rated. The quality of digital images given to students via Universal Serial Bus (USB) flash drives and over the internet was appreciated. The almost universal complaints were the perceived poor readability of the textbook and the feeling that in-person quizzes were unnecessary. The most popular segment of the course was the online review of select imaging cases with the radiologist. The need for time away to interview for residency positions was highly valued, and the ability to perform online readings and modules allowed the students the freedom to travel during the radiology course while staying up with the course content (Textbox 1).

Mean final exam scores from the AMSER 80-question online quiz test bank showed no significant changes based upon the trimester that the radiology course was taken (Table 1).

**Student feedback on the online modules.**

Positive student feedback

- Hands-on ultra-sound workshop was motivating
- Flexibility for time away from school allowed low-stress interview travel
- CORE series of modules from Aquifer were excellent for learning
- Self-instruction independence was appreciated
- Webinars with radiologists allowed efficient understanding of didactics
- Final exam from The Alliance of Medical Student Educators in Radiology was fair

Negative student feedback

- Quizzes felt unnecessary and should be eliminated
- Textbook was poor and did not align well with online didactics
- Time with imaging techs was wasted and should be eliminated
- The American College of Radiology app tables are tedious to review
- Opportunities to shadow with radiologists should be available

**Table 1:** Mean final exam scores (N=40).

Months	n (%)	Mean % score	*P* value^a^
July, August, and September	6 (15)	73.3	.30
October, November, December, and January (interview months)	26 (65)	69.2	N/A^b^
February, March, and April	8 (20)	72.6	.34
Total	40 (100)	70.5	N/A^b^

^a^*P* values calculated as the given period versus the October to January period.

^b^N/A: not applicable.

## Discussion

### Principal Findings

Traditional instruction in imaging and radiology requires 2 to 4 weeks of time, with the largest quantity of time spent shadowing a practicing radiologist in a reading room. Understated in the teaching of radiology are the huge number of normal findings that are seen and the rarity of abnormal findings. This learner shadowing technique is therefore time-intensive and utilizes “spoon-feeding” of seldom-seen pathologic findings found within normal findings and common maladies. Efficiently reading undifferentiated images amidst the time-restraints of modern medical group economics can adversely impact the time available for the faculty to teach. As faculty pay has become more heavily influenced by productivity bonuses, limitations of time can result in more spoon-feeding of students. This may, to some extent, compromise the time required for the students to learn to think independently and critically.

Despite the drawbacks of the traditional “analog” method of shadowing radiologists, many students still enjoy this largely passive traditional adventure. The lifestyle attributes and shift work of a radiologist, the escape from the stresses of inpatient care, and the sub-optimal effort required when being “spoon-fed” take less time each day [7] than the long call schedules of internal medicine or surgery. Students have competing urgencies, so it is little wonder this important part of their education is too often referred to as a “radiation vacation.”

Student’s preference for this passive “spoon feeding” makes sense from their point of view. Today’s students interview at an average of 13.3 residency programs [2] and travel takes time and money. Students want, and perhaps need, more time off to visit the residency programs of their choice. Radiation vacations may be the best or only travel-friendly rotation available in some educational programs, but it may not deliver the best educational experience for the 96.5% of students who will not go into radiology.

Medical student satisfaction in German medical school subinternships showed increased student satisfaction when students were given increased academic teaching, personal involvement in learning, and more practical skills [8]. In US medical schools, students enjoy hands-on Point-of-Care ultrasound training, and believe it to be educationally useful [9]. It is clear that students in the United States are strapped for time, desire freedom, and benefit from a more independent, yet hands-on learning experience in radiology.

We accept that physicians order imaging tests without communicating with a radiologist prior to entering such an order. It is of interest, then, that 77% of medical students have not heard of the ACR Appropriateness Criteria [10] nor have they used the free ACR online app. We have an educational imperative to teach ordering physicians of the future to navigate the most effective path in their pursuit of their patients’ health. We present a modular, Web-based approach, with online basic sciences and radiologic anatomy lessons preceding online selected cases. This order is similar to the method used by Ertl in which imaging technology, anatomy, side effects, risks, and content precede diagnostic decision-making and imaging [11]. Indeed, imaging diagnostics do not start the training session. Traditional radiology training focused on “spoon-feeding” (shadowing experiences) where information flowed from teacher to learner [12]. Efforts to increase educational retention and student satisfaction have been successful with activities that allow independent problem-solving, investigation, and discovery. Outcomes from such online learning programs have yielded higher learner performances in practicing evidence-based-medicine and in patient management skills [13]. Self-paced themes with faculty feedback increase student confidence and knowledge in radiology [14]. Peer-to-peer learning is highly appreciated with good outcome studies noted in teaching and learning Point-of-Care ultrasound [15]. The mix of formal online didactics, required online readings, online modules, and a de-emphasis on shadowing can improve the educational outcome of the student, increase student satisfaction, and unload the burden on the workflow of the faculty radiologist [16].

Students are still 4 to 8 years away from entering practice. It follows that their education should include training for the medical practice of the foreseeable future. New technology has delivered ultrasound images to our electronic tablets and phones. The changes in practice models have placed these compact transducers and screens into our emergency rooms and community clinics. In Peru and Nepal, a 7-day course in diagnostic ultrasound taught general outpatient practitioners to diagnose pneumonia in children with improved sensitivity and specificity compared to the World Health Organization algorithm [17]. The ultrasound exam took an average of 6.4 minutes. At a non-university hospital in Norway, cardiac and abdominal Point-of-Care ultrasounds took 5.7 and 4.7 minutes, respectively [18]. The results led to major changes in the diagnosis of 6.5% of patients, and added additional important diagnoses in 24%.

We present a novel, largely online medical school imaging curriculum with a focus on utilization of imaging services as well as diagnostics. We believe this approach would be readily accepted by a majority of medical students who need to be away from the radiology reading rooms during interview or travel months, yet also wish to learn to efficiently and safely order imaging tests, review basic digital images, and use basic ultrasound equipment. We would argue that, with over 96% of students entering fields other than radiology, this internet-based approach could be adapted to a majority of medical colleges.

The mix of modalities, including assigned online textbook chapters, online commercial modules, video training vignettes, and teleconference didactic presentations provide for engaged learning. This is in contrast to shadowing or “spoon-feeding.” The use of didactic methods that optimize Web-based independent learning makes sense for the majority of students whose priorities align with the realities of increased competitiveness for residency spots. Students can be away from campus for 4th year residency interviews and watch online videos from their hotel rooms, or while on a plane. Students may unfortunately choose “easy” clerkship electives based upon the ability to travel away from the medical school site and interview. This approach embraces the “radiation vacation” and encourages its use between the months of October and January. The absence of statistical difference in AMSER testing results from students who took the course before (*P*=.30) or after (*P*=.34) the interview or travel season confirms that medical knowledge learning outcomes showed no differences due to taking the course during the residency interview season. Additionally, students were pleased that they were allowed to be away from the university while continuing to study via their online links, online modules, and hyperlinked online textbook assignments. Online videoconferences with the radiology faculty received universal praise for effectiveness and enjoyment. Out-of-town travel did not degrade learning outcomes.

The use of evening faculty case presentation via Bluejeans or Google Hangout software allowed back-and-forth discussion of specific cases. Cases were individualized according to the lesson and the level of learning. This is in contrast to traditional shadowing where cases include random diagnoses, are often taken in the order the staff radiologist receives them, and are dictated in a manner respectful of radiology departmental efficiency. Teaching takes time and planning. The online curriculum avoids the pitfalls of shadowing, embraces the absenteeism inherent with the interview season, and teaches to the level of the curricular outcomes.

The peer-taught, hands-on ultrasound curriculum also received universally positive feedback. Student engagement was optimized by requiring each student to lead and facilitate a problem-based-learning presentation to the group of a clinical case using digital imaging. In contrast, the experience of following imaging techs in the hospital received negative feedback due to its passive shadowing nature. This was consistent with findings by others [19], therefore it was halted.

While our learning objectives (Multimedia Appendix 1) and mission may not precisely align with those of the traditional radiology subinternship, we found that scores on the nationally administered AMSER examination (70.5%) were in line with the national AMSER 20-question results (70%).

The capacity for lifelong learning in an environment of constant change is enhanced by our use of independent Web-based learning. Cost-effectiveness, side-effects, adverse outcomes, and unnecessary interventions related to false-positives and false-negatives are presented in a progressive and integrated manner due to the ability to control the presentation of online pathology during the curriculum. Use of the mobile app from the ACR prior to ordering imaging studies is taught and reinforced throughout the course. Indeed, one of the complaints of the students related to the tedious nature of continually looking up the ACR Appropriateness Criteria. Despite the students’ complaints about the repetition of referring to the ACR Appropriateness Criteria, they were grateful that the ACR created such an app.

### Conclusion

Students of the 21st century require and demand increased time to interview for residency positions. We have co-opted the traditional “radiation vacation” with online delivery of a mixed-modality Web-centered radiology experience that can be performed with substantial absenteeism from the physical medical school environment. Despite an absence of direct onsite radiologist shadowing and mentoring, AMSER testing outcomes were comparable. The course emphasis on the importance of evidenced-based imaging utilization and teleconference reviewing of specific case films with a paucity of in-person shadowing did not alter the students’ course satisfaction. An effective internet-based imaging course which acknowledges course objectives, the immediate needs of the students, and those of their future patients can be taught to 21st century medical students.

## Appendix

Multimedia Appendix 1 Curriculum of Web-based radiology course.

## Abbreviations

ACR: American College of Radiology
AMSER: The Alliance of Medical Student Educators in Radiology

## References

[1] Poot JD, Hartman MS, Daffner RH (2012). Understanding the US medical school requirements and medical students' attitudes about radiology rotations. Acad Radiol.

[2] ResultsData 2MRM The National Residency Matching Program.

[3] Shi J Diagnostic Imaging.

[4] Gispen F, Magid D (2016). Assessing Medical Student Knowledge of Imaging Modality Selection Before and After a General Radiology Elective: A Comparison of MS-IIs, MS-IIIs, and MS-IVs. Acad Radiol.

[5] Gunderman R (2014). Essential Radiology Clinical Presentation Pathophysiology Imaging Third Edition. New York, NY. Thieme.

[6] Case-Based Online Radiology Education (CORE) Aquifer Radiology.

[7] DeLong Suzanne Teaching Methods to Encourage Independent LearningThinking.

[8] Kasch R, Wirkner J, Hosten N, Hinz P, Napp M, Kessler R (2016). Subinternship in Radiology - A Practical Start to the Specialization?. Rofo.

[9] Webb EM, Cotton JB, Kane K, Straus CM, Topp KS, Naeger DM (2014). Teaching point of care ultrasound skills in medical school: keeping radiology in the driver's seat. Acad Radiol.

[10] Prezzia C, Vorona G, Greenspan R (2013). Fourth-year medical student opinions and basic knowledge regarding the field of radiology. Acad Radiol.

[11] Ertl-Wagner B, Barkhausen J, Mahnken AH, Mentzel HJ, Uder M, Weidemann J, Stumpp P, German Association of Chairmen in Academic Radiology (KLR), German Roentgen Society (DRG) (2016). White Paper: Radiological Curriculum for Undergraduate Medical Education in Germany. Rofo.

[12] Rahim S, Ros P (2016). Moving Away From Spoon-Feeding as a Teaching Style in Radiology. AJR Am J Roentgenol.

[13] Wiecha J, Vanderschmidt H, Schilling K (2002). HEAL: an instructional design model applied to an online clerkship in family medicine. Acad Med Sep.

[14] Lim-Dunham JE, Ensminger DC, McNulty JA, Hoyt AE, Chandrasekhar AJ (2016). A Vertically Integrated Online Radiology Curriculum Developed as a Cognitive Apprenticeship: Impact on Student Performance and Learning. Acad Radiol.

[15] Naeger D, Conrad M, Nguyen J, Kohi M, Webb E (2013). Students teaching students: evaluation of a “near-peer” teaching experience. Acad Radiol.

[16] Hilmes M, Hyatt E, Penrod C, Fleming A, Singh S (2016). Radiology in Medical Education: A Pediatric Radiology Elective as a Template for Other Radiology Courses. J Am Coll Radiol.

[17] Chavez MA, Naithani N, Gilman RH, Tielsch JM, Khatry S, Ellington LE, Miranda JJ, Gurung G, Rodriguez S, Checkley W (2015). Agreement Between the World Health Organization Algorithm and Lung Consolidation Identified Using Point-of-Care Ultrasound for the Diagnosis of Childhood Pneumonia by General Practitioners. Lung.

[18] Andersen GN, Graven T, Skjetne K, Mjølstad OC, Kleinau JO, Olsen O, Haugen BO, Dalen H (2015). Diagnostic influence of routine point-of-care pocket-size ultrasound examinations performed by medical residents. J Ultrasound Med.

[19] Chen JY, Lewis PJ (2014). The value of a medical student radiology triage program in enhancing clinical education and skills. Acad Radiol.

