# Supplementary material for: An Internet-Based Radiology Course in Medical School: Comparison of Academic Performance of Students on Campus Versus Those With Absenteeism Due to Residency Interviews
Source: JMIR Med Educ. 2018 May 18;4(1):e14. doi: 10.2196/mededu.8747 (PMC5984272; doi:10.2196/mededu.8747)
Supplement: Multimedia Appendix 1 [file mededu_v4i1e14_app1.pdf]

Radiology Rotation

Update 3-4-17

Objective: Through independent required reading assignments, Med-U CORE modules, on-line scenarios, hands-on technical instruction, inter-professional association with technicians, and practice reading sessions with radiologists, students will learn to incorporate evidence-based strategies for imaging services, while experiencing the art, science, and diversity of modern imaging.

Required Textbook: Learning Radiology Recognizing the Basics 3rd Edition by William Herring. Cost used is $47.50 (Amazon). Rent for $19.00. Read chapters one and two prior to day one of class and bring it to day #1 of your clerkship rotation.
Also required: A Laptop computer capable of taking a USB flash drive and accessing the UCR SOM library by internet. Bring your laptop to class on the first day.

Presentations: Each student will present an actual case history (with HIPAA information redacted) along with associated imaging studies. The student will lead the Problem-Based Learning discussion through the differential diagnosis to its conclusion, using ACR criteria for appropriateness and actual images with interpretations. This will represent 20% of the final course grade.

Evening or weekend classes are important. You will be involved in internet-based small group sessions with our radiology content specialists.

Final Examination: The final exam is a national shelf exam. It includes image-based questions, as well as questions without images regarding anatomy, appropriate ACR imaging management, and other general principles of diagnostic imaging. The final exam is based on material covered in modules, assigned links, chapters from Herring, and lectures.

Student Evaluations and grading:

- MRI and Radiation Safety Quiz (Passing is Mandatory)
- A 30 minute student presentation on a self-selected case history presentation during the final week (20%)
- Course quizzes (measuring completion of required readings and modules) (30%)
- Radiologist and Technician Evaluations of Professionalism, Communications Skills, Medical Knowledge, and Systems-Based Knowledge (20%)
- Final examination consisting of questions based on information and images covered in lecture material 30%)

- Final grades in excess of 85% are eligible for honors if no professionalism issues are encountered

Radiology Rotation Goals and Objectives

Knowledge for Practice

1. Know critical and high priority imaging findings and diagnoses and understand basic interpretive techniques in each subspecialty area.

2. Know the indications for the most important imaging examinations in each of the Radiology subspecialty areas.

3. Demonstrate knowledge of human anatomy by recognizing key structures on various imaging modalities in each of the Radiology subspecialty content areas.

Patient Care (Problem Solving and Clinical Skills)

1. Regard the critical importance of useful clinical history in imaging interpretation

2. Recognize the consequences of radiation in humans of different genders and ages

3. Understand the effects of radiographic dye on patients with kidney disease

Practice-Based Learning and Improvement

1. Describe the common imaging findings of at least one pathologic entity, present an imaging differential diagnosis of these findings, and demonstrate understanding of the appropriate imaging evaluation and involved pathophysiology.

Systems-Based Practice

1. Understand the role of the radiologist in the care of patients undergoing imaging evaluation and/or image guided procedures or for whom such evaluation or procedures are being considered.

2. Know the relative costs associated with radiologic testing

3. Understand the role that false positive and false negative results from mammography have on recommendations for screening

Interpersonal and Communication Skills

1. Effectively advise patients and colleagues on the risks, benefits, limitations and indications of each of the most common imaging examinations.

2. Demonstrate understanding of the important role of communication in radiology with specific emphasis on the radiology report, urgent or unexpected findings, recommendations for follow-up imaging or procedures, and doctor patient communication.

Professionalism

1. Demonstrate understanding of the principles of mutual respect, honesty, and discretion in the use of patient clinical and imaging data, during lecture, as a part of the clinical radiology team, and when interacting with referring clinicians and non-radiology colleagues and support staff.

Interprofessional Collaboration

1. Demonstrate the ability to engage in an Interprofessional team in a manner that optimizes safe, effective patient and population-centered care.

Personal and Professional Development

1. Demonstrate trustworthiness that makes colleagues feel secure when one is responsible for the care of patients

Course Description (Read Chapters 1 and 2 **prior to the first day** of class.)

Day 1 AM: Intro to Radiology: ½ day didactic with ½ day interactive learning activities. You should have read Chapters 1 and 2 **prior to the first day** of class. Assigned readings in Learning Radiology Recognizing the Basics 3rd Edition, by William Herring, as well as assigned modules and online lessons, are mandatory: Also mandatory: a laptop with the ability to accept a USB flash drive with access to the UCR SOM library Access Medicine site. Bring your book and computer with you to classes on the days when there are assigned lessons.

Site: Classroom at UCR (the 4th year coordinator will have the exact room)

A. Reference Text reading assignment: Learning Radiology 3rd Edition by William Herring. Pre-read Chapters 1 and 2 for Day #1. Read chapter 3 and 4 prior to day #2.
B. Assigned readings for MRI and Radiation Safety (quiz on day 2): http://www.imagewisely.org/imaging-modalities/computed-tomography/imaging-physicians/articles/ionizing-radiation-effects-and-their-risk-to-humans. http://www.radiologyinfo.org/en/info.cfm?pg=safety-xray#safety-radiation-dose
C. Terminology used: Fluoroscopy, ultrasound, Tomogram, CT, CTA, Subtraction angiography, MRA, MRI, Nuc Med, PET. Coronal, Sagittal, Axial, Contrast, Gadolinium, Technetium,

Sestamibi, Transducer, Sievert, Gray, Rad, Rem, T1 and T2 weighted spin, Window, Reconstruction, anechoic, echogenic, hypoechoic. Students will define these as a group.

D. **Quiz on terms** (in C above)
E. Afternoon rotating assignments/activities to be announced

Day 1 PM: Online activities only

1. Introduction. http://www.dartmouth.edu/~anatomy/HAE/Radiology_Intro/rad/radintro.html

2. How images are obtained
http://www.dartmouth.edu/~anatomy/HAE/Radiology_Intro/rad/rad1/rad1a.html

3. Factors affecting appearance
http://www.dartmouth.edu/~anatomy/HAE/Radiology_Intro/rad/rad2/rad2a.html

4. What structures are well seen on radiographs?
http://www.dartmouth.edu/~anatomy/HAE/Radiology_Intro/rad/rad3/rad3a.html

5. Indications for use
http://www.dartmouth.edu/~anatomy/HAE/Radiology_Intro/rad/rad4/rad4a.html

6. Chest Radiography Primer
http://www.med-ed.virginia.edu/courses/rad/cxr/index.html
7. How to search a CXR video
https://www.youtube.com/watch?v=HfNU8DGXFgk

Read Chapters 3 and 4 in Learning Radiology 3rd Edition prior to Day #2

Day 2 AM: Principles of Radiation. (You should have read Chapters 3 and 4 in Learning Radiology 3rd Edition prior to Day #2)

Site: Classroom at UCR

A. Definitions of
B. Ionizing radiation effects on the human body, fetal to adult
C. Radiation physics
D. Grids, Distance, Scatter
E. Quiz on Radiation Safety, video content, and Herring Chapters 1-4.

Day 2 PM: Pneumonia, Pleural Effusions, ARDS.

1. CORE Med-U.org assignment: Module #1.
2. Reading a CXR: http://www.med-ed.virginia.edu/courses/rad/cxr/index.html
3. Website necessary for the course = https://acsearch.acr.org/list (If you put the APP on your phone you can use it during the quizzes).

Read Chapters 5 & 6 prior to day #3.

Day **#3** AM: Pre-op screening, Pulmonary nodules, Pneumothorax, lung cancer, pneumonectomy.

A. CORE Med-U.org Module #2
B. CT, CT angiogram of the lung and CT Chest anatomy tutorial
   http://www.med-ed.virginia.edu/courses/rad/CTChestAnatomy/CTAnatomyoftheChest.htm

Day 3 PM: **Quiz on CXR anatomy**

Read chapters 7, 8, 9, and 10 in Herring prior to Day #4.

Day #4 AM: The Chest. AM: emphysema, aortic dissection, ICU Lines and Tubes, CHF, PE.
Day #4 PM: half of students will shadow Radiology Techs

A. CT Chest anatomy tutorial, continued
   http://www.med-ed.virginia.edu/courses/rad/CTChestAnatomy/CTAnatomyoftheChest.htm
B. CT interpretation using windows and reconstructions video
   https://www.youtube.com/watch?v=4pb1f79h7_I&feature=youtu.be
C. Med-U.org module #4: Chest Medicine
D. Read Herring Chapters 11, 12, and 13.

Be prepared for a quiz on Day #5.

Day 5 AM: **Quiz on Chest Anatomy, Herring Chapters 2-13 and CORE module assignments**

Day 5 PM: Free study time

Day #6 of class

AM: Half of students will shadow hospital radiology techs

PM: Perform CORE module 5. Reference https://acsearch.acr.org/list for each situation you covered in the module.

Read chapters 14, 15, and 16 in Herring prior to the next class.

Day #7 of class: Normal and abnormal abdominal radiology findings, including plain films and CT.

AM: 1. Perform CORE module 6
2. Reference https://acsearch.acr.org/list for each situation you covered in the module.

PM: 1. Read Herring Chapter 20 and prior to Day #8.

Day #8, abdomen.

1. Perform CORE module #8
2. Reference https://acsearch.acr.org/list for each situation you covered in the module.

Read Herring Chapter 21 prior to Day #9

Day #9, abdomen and u/s

3. Hands-on ultrasound clinic.
   a. Bladder scanning
   b. Gall bladder and CBD ultrasound

Read Herring Chapter 17 and 21 prior to day #10

Day #10 of class: Ultrasound in medicine, continued.

1. Perform CORE module #7
2. Reference https://acsearch.acr.org/list including Venous Duplex and Arterial Duplex

PM: Quiz on ultrasound, Herring readings, CORE modules, and ACR Appropriateness Criteria

Day #11 PM: Skeletal Trauma:

1. Read chapter 24.

2. Review your skeletal anatomy (http://uwmsk.org/RadAnatomy.html))

3. **Review http://emedicine.medscape.com/article/104363-overview .**
4. Read the "Fractures" subchapter of Chapter 10 up to but not including the subsection paragraphs that begin with the subtitle, "Lower Extremity Fractures" in the online text Current

Diagnosis & Treatment Orthopedics 5th Edition The Salter classification and upper extremity fractures: http://accessmedicine.mhmedical.com/content.aspx?sectionid=45451716&bookid=675&jumpsectionID=45457972&Resultclick=2

Read chapter 19 prior to the next class and perform CORE module # 3

Day #12 of class, AM: chest trauma

AM: Perform CORE module #16

PM: Shadow hospital radiology

Read Herring Chapters 22 and 27 prior to the next day

Day #13: CT and MRI

AM: Perform CORE Module #9

PM: Dr. Millard Rounds

Read Herring Chapter 26 prior to the next day

Day #14: Potpourri of diagnoses

AM: Perform CORE Modules #10 and #13

PM: Dr. Millard Rounds

Read Herring Chapter 28 prior to the next day

Day #15: Pediatrics

AM: Perform CORE Modules 11 and 12.

PM: Review time

Day #16: Childhood trauma and Woman's imaging

AM: 1. Read the "Fractures" subchapter of Chapter 10 up to but not including the subsection paragraphs that begin with the subtitle, "Lower Extremity Fractures" in the online text Current Diagnosis & Treatment Orthopedics 5th Edition. The Harris-Salter classification and upper extremity fractures are a rich source of board questions: http://accessmedicine.mhmedical.com/content.aspx?sectionid=45451716&bookid=675&jumpsectionID=45457972&Resultclick=2

2. Perform CORE Module #14

PM: Dr. Millard Rounds

Day #17: Woman's imaging

AM: Perform CORE Module #15

PM: Moose & Doc Breast Cancer http://breast-cancer.ca/ultrahypo-echosolid/ , http://breast-cancer.ca/2a-screening/

Day #18

AM: Student roundtable with USB drive cases

PM: Student Reports/Student-led Radiology Problem-Based Lessons (PBL) Cases.

Day #19:

AM: Continuation of Student Reports/Student-led Radiology Problem-Based Lessons (PBL) Cases.

PM: Independent study time for final exam

Day #20: Final Exam

The Alliance of Medical Student Educators in Radiology (AMSER) prepares an 82-question AMSER national shelf exam. This will be taken online, in a fashion similar to all other NBME/USMLE examinations. Grading will be on a national curve.
